# pH-Responsive Nanostructures Based on Surface Active Fatty Acid-Protic Ionic Liquids for Imiquimod Delivery in Skin Cancer Topical Therapy

**DOI:** 10.3390/pharmaceutics12111078

**Published:** 2020-11-11

**Authors:** Silvia Tampucci, Lorenzo Guazzelli, Susi Burgalassi, Sara Carpi, Patrizia Chetoni, Andrea Mezzetta, Paola Nieri, Beatrice Polini, Christian Silvio Pomelli, Eleonora Terreni, Daniela Monti

**Affiliations:** 1 Department of Pharmacy, University of Pisa, Via Bonanno 6, 56127 Pisa, Italy; susi.burgalassi@unipi.it (S.B.); sara.carpi@unipi.it (S.C.); patrizia.chetoni@unipi.it (P.C.); andrea.mezzetta@unipi.it (A.M.); paola.nieri@unipi.it (P.N.); beatrice.polini@farm.unipi.it (B.P.); christian.pomelli@unipi.it (C.S.P.); eleonora.terreni@farm.unipi.it (E.T.); daniela.monti@unipi.it (D.M.); 2 NEST, Istituto Nanoscienze-CNR and Scuola Normale Superiore, Piazza San Silvestro 12, 56127 Pisa, Italy

**Keywords:** ionic liquids, nanostructures, imiquimod, skin cancer, topical drug delivery

## Abstract

For topical treatment of skin cancer, the design of pH-responsive nanocarriers able to selectively release the drug in the tumor acidic microenvironment represents a reliable option for targeted delivery. In this context, a series of newly synthesized surface-active fatty acid-protic ionic liquids (FA-PILs), based on tetramethylguanidinium cation and different natural hydrophobic fatty acid carboxylates, have been investigated with the aim of developing a pH-sensitive nanostructured drug delivery system for cutaneous administration in the skin cancer therapy. The capability of FA-PILs to arrange in micelles when combined with each other and with the non-ionic surfactant d-α-Tocopherol polyethylene glycol succinate (vitamin E TPGS) as well as their ability to solubilize imiquimod, an immuno-stimulant drug used for the treatment of skin cancerous lesions, have been demonstrated. The FA-PILs-TPGS mixed micelles showed pH-sensitivity, suggesting that the acidic environment of cancer cells can trigger nanostructures’ swelling and collapse with consequent rapid release of imiquimod and drug cytotoxic potential enhancement. The in vitro permeation/penetration study showed that the micellar formulation produced effective imiquimod concentrations into the skin exposed to acid environment, representing a potential efficacious and selective drug delivery system able to trigger the drug release in the tumor tissues, at lower and less irritating drug concentrations.

## 1. Introduction

The incidence of skin cancer is on the rise worldwide and surgical removal or invasive procedures represent the most widely used treatments. In recent years, numerous therapeutic strategies have been introduced, such as immunotherapy, targeted therapy, photodynamic therapy, and gene therapy [1,2,3,4]. While non-melanoma skin cancer is rarely fatal, melanoma can metastasize and account for most of the skin cancer deaths. The primary treatment for melanoma is surgical, but non-surgical treatments are employed as adjuvant therapies and in those cases where excision is not feasible or prior resections have been unsuccessful [2,5,6,7,8]. In particular, in case of unresectable melanoma, in-transit metastatic melanoma, and lentigo maligna, non-surgical approaches can be pursued and include systemic agents, local radiation, and intralesional and topical therapies [8,9]. Moreover, with advances in nano-technology and drug delivery systems, skin cancer treatment has been diversified and nanostructure-based formulations represent promising options, especially for topical delivery [10,11,12]. The topical application of drugs represents one of the most preferable routes for drug administration because of advantages as first pass metabolism bypass and increased patient compliance [13]. For the last few decades, the nanotechnologies have been studied to enhance drug bioavailability following topical application [10,14,15,16,17] and received increasing attention for the treatment of melanoma [2,7,18]. Still, the field is open for the development of more potent and biocompatible topical formulations.

The new strategies for drug development include the use of existing drugs in the formulation of sustainable and biodegradable nanosystems for specific target delivery. This approach has the potential for reducing time and costs of developing new drugs for clinical use. However, the controlled release of the drug from the nanostructures represents a key point in the architecture of new drug delivery systems, taking into account that drug release can be influenced by many factors [14,19]. In order to design an efficient drug delivery system, polymeric structures able to respond to environmental stimuli characteristic of the tumor tissue (temperature, pH, redox properties, enzyme activity) to activate drug release, have been widely investigated [19]. Among these, pH-responsive nanosized carriers are able to selectively release the drug in the tumor acidic microenvironment or under the acidic endo/lysosome condition of cancer cells [20,21] by exploiting either the capability of some polymers with ionizable groups to be protonated at acidic pH or the performance of polymers containing acid-labile linkages to undergo cleavage of the bonds and consequent chain degradation [22]. In this context, prodrug hybrid micelles [21], nanogels [20,23], and nanomicelles [19] have been proposed.

Ionic liquids (ILs) are organic salts with melting points below 100 °C which are endowed with unique physico-chemical properties such as low flammability, negligible vapor pressure, high thermal and chemical stability, and wide electrochemical window [24,25,26,27,28]. By careful selection of the constituting ions, the features of interest of an IL can be tailored to a given application. The range of potential applications of ILs span from alternative solvents for organic reactions [29] to dissolution media for biopolymers [30], and from lubricants [31] to electrolyte for batteries [32], to mention a few. Undeniably, in the last few years, the biomedical and pharmaceutical industry has turned increasing attention towards ILs due to their yet unexplored potential as bioactive agents or to their ability to solubilize and stabilize drugs [33,34,35,36,37,38,39]. Furthermore, the cation and/or anion amphiphilic nature can be finely balanced to trigger the self-assembling behavior of ILs [40,41,42,43,44]. In this framework, fatty acids raised remarkable interest as building blocks for ILs containing natural hydrophobic tails [45,46,47,48,49,50] and such materials were investigated in pharmaceutical applications [42,51,52,53,54]. An intriguing subclass of ILs is that of the so-called protic ionic liquids (PILs), which are easily prepared by reacting a Brønsted acid and a Brønsted base [55,56].

**Figure 1:**
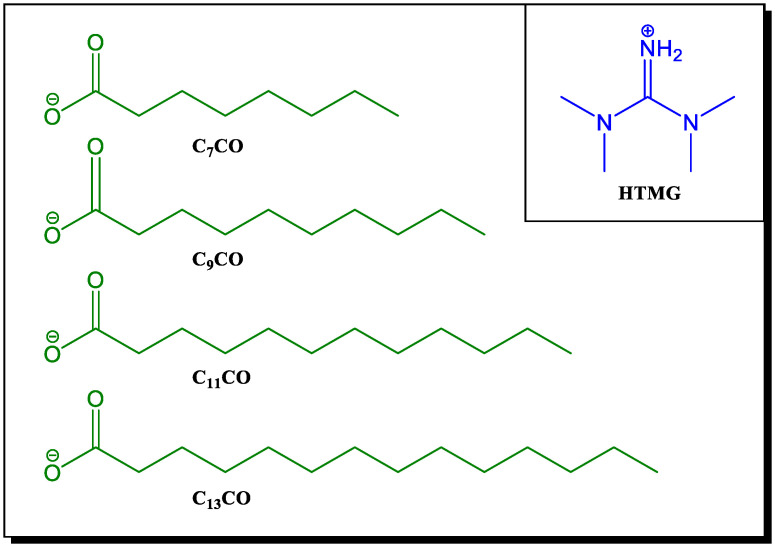
Carboxylate anions (FA, in green), obtained from the natural fatty acids, and tetramehyl guanidinium cation (in blue) components of the prepared fatty acids protic ionic liquids (FA-PILs).

**Scheme 1:**
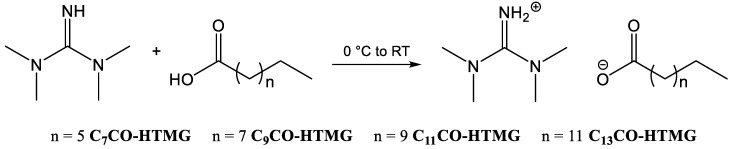
Synthesis of C_n_CO-HTMG.

The objective of the present work was to demonstrate the capability of a series of newly synthesized fatty acid protic ionic liquids (FA-PILs, C*n*CO-HTMG; (Figure 1 and Scheme 1), based on the tetramethylguanidinium cation (HTMG) and different natural hydrophobic fatty acid carboxylates (FA, C*n*CO) to behave as surfactants and form micelles with dimensions in the nanometer range. The ultimate aim was to develop a pH-sensitive nanostructured drug delivery system for cutaneous administration of drugs against skin cancer. Moreover, the effect of FA-PILs on the physico-chemical properties of a non-ionic aqueous surfactant, such as d-α-Tocopherol polyethylene glycol succinate (vitamin E TPGS), has been evaluated. Indeed, it has been reported that surfactants are able to form various molecular aggregates in ILs [57], similar to micellization or liquid crystals’ formation in aqueous media.

In this context, mixtures of vitamin E TPGS and FA-PILs in water to investigate micelle formation have also been studied in order to shed light on the formation of potentially novel micellar structures. Vitamin E TPGS was chosen for its multiple properties as a potent solubilizer, drug permeation enhancer, and for its ability to overcome multi-drug resistance (MDR) by inhibiting P-glycoprotein (P-gp), thus resulting in an anticancer agent [21,58].

Besides, the new drug delivery systems should improve drug-selective tissue distribution in the basal layer region, minimizing side effects and reducing the required dose. 

Therefore, they have been proposed for the delivery of imiquimod (IMQ), an immuno-stimulant drug topically used for the treatment of skin and mucosal diseases [59,60]. Although not yet approved for the treatment of melanoma, many off-label studies have suggested IMQ as a beneficial treatment for this type of cancer [61,62,63,64], and the Italian Association of Medical Oncology (AIOM) guidelines (2019) have confirmed its efficacy in lentigo maligna (a particular type of melanoma) therapy. The development of formulations containing IMQ and its delivery to the skin is not easy due to its poor solubility both in water and in many of the excipients used in the pharmaceutical field and to poor skin penetration properties [16]. The use of nanotechnologies could improve drug solubility and contemporaneously increase the drug concentration at the target site by overcoming the stratum corneum, i.e., the main barrier to penetration [60]. In the present study, pH-sensitive self-assembling micelles, based either on FA-PILs or FA-PILs/Vitamin E TPGS and containing IMQ, were tuned up. The self-assembling behavior and pH-triggered drug release in response to acidic tumor environment, as well as the in vitro cytotoxicity of the nanostructures on 501Mel melanoma cells, were investigated. Finally, the drug penetration profile into the skin layers after cutaneous application of the prepared micelles was studied through in vitro permeation/penetration experiments using porcine ear skin as a model.

## 2. Materials and Methods 

### 2.1. Materials

The following materials were used: d-α-Tocopherol polyethylene glycol succinate (VitE-TPGS, Kolliphor^®^TPGS, BASF, Ludwigshafen, Germany), and 1-(2-methylpropyl)-1H-imidazo[4,5-c] quinolin-4-amine (Imiquimod, IMQ, Tocris Bioscience, Bristol, UK).

All other reagents were analytical grade. Ultra-pure water was prepared using Milli-Q^®^ plus apparatus (Millipore, Milan, Italy).

Tetradecanoic acid (98%) was obtained from Alfa Aesar. (Kandel, Germany), 1,1,3,3-tetramethylguanidine (99%), dodecanoic acid (≥99%), decanoic acid (≥99.5%), and octanoic acid (≥99%) were purchased from Sigma Aldrich (Darmstadt, Germany).

### 2.2. Preparation and Characterization of Fatty Acid-Protic Ionic Liquids (FA-PILs)

#### 2.2.1. Preparation of FA-PILs

An equimolar amount of fatty acid was added to 1,1,3,3-tetramethylguanidine at room temperature, without any solvent addition. The reaction mixture was heated to 60 °C and stirred for 1 h. At the end of the reaction, the mixture was cooled to room temperature to obtain a white solid product. The obtained product was analyzed by ^1^H NMR.

Tetramethylguanidinium octanoate C_7_CO-HTMG. The preparation of C_7_CO-HTMG (99% yield, white greasy solid) was performed according to the general procedure. ^1^H NMR (CDCl_3_) 8.80 (bs, 2H, N*H*_2_^+^), 2.89 (s, 12H, 4 × N-C*H*_3_), 2.07 (t, 2H, C*H*_2_COO^−^), 1.55–1.45 (m, 2H, C*H*_2_CH_2_COO^−^), 1.25–1.09 (m, 8H, 4 × C*H*_2_ chain), 0.78 (t, 3H, CH_3_).

Tetramethylguanidinium decanoate C_9_CO-HTMG. The preparation of C_9_CO-HTMG (99% yield, white solid) was performed according to the general procedure. ^1^H NMR (CDCl_3_) 8.35 (bs, 2H, N*H*_2_^+^), 2.95 (s, 12H, 4 × N-C*H*_3_), 2.16 (t, 2H, C*H*_2_COO^−^), 1.65–1.49 (m, 2H, C*H*_2_CH_2_COO^−^), 1.34–1.16 (m, 8H, 4 × C*H*_2_ chain), 0.86 (t, 3H, CH_3_).

Tetramethylguanidinium dodecanoate C_11_CO-HTMG. The preparation of C_11_CO-HTMG (99% yield, white solid) was performed according to the general procedure. ^1^H NMR (CDCl_3_) 8.83 (bs, 2H, N*H*_2_^+^), 2.89 (s, 12H, 4 × N-C*H*_3_), 2.09 (t, 2H, C*H*_2_COO^−^), 1.58–1.44 (m, 2H, C*H*_2_CH_2_COO^−^), 1.28–1.09 (m, 8H, 4 × C*H*_2_ chain), 0.81 (t, 3H, CH_3_).

Tetramethylguanidinium tetradecanoate C_13_CO-HTMG. The preparation of C_13_CO-HTMG (99% yield, white solid) was performed according to the general procedure. ^1^H NMR (CDCl_3_) 8.13 (bs, 2H, N*H*_2_^+^), 2.94 (s, 12H, 4 × N-C*H*_3_), 2.15 (t, 2H, C*H*_2_COO^−^), 1.55–1.48 (m, 2H, C*H*_2_CH_2_COO^−^), 1.36–1.11 (m, 8H, 4 × C*H*_2_ chain), 0.86 (t, 3H, CH_3_).

#### 2.2.2. NMR Analysis

^1^H and ^13^C NMR spectra were recorded with a Bruker Avance II operating at 400 MHz at 24 °C. For the first-order proton chemical shifts CDCl_3_ (H 7.26, C 77.16), the chemical shifts are given in δ. The following abbreviations are used: s = singlet, m = multiplet, bs = broad singlet, t = triplet, bt = broad triplet, q = quartet, qui = quintuplet, sext = sextet.

#### 2.2.3. Differential Scanning Calorimetry (DSC)

The thermal behavior of the ionic liquids was analyzed by a differential scanning calorimeter (TA DSC, Q250, New Castle, USA, Delaware, temperature accuracy ± 0.05 °C, temperature precision ± 0.008 °C, enthalpy precision ± 0.08%) following the procedure reported in Mezzetta et al. [50]. After drying at 120 °C for 30 min, the phase behavior was explored under nitrogen atmosphere in the temperature range of −90–200 °C with different heating rates (ν_h_ = 2–20 °C/min). DSC experiments were carried out in duplicate.

#### 2.2.4. Surface Tension Measurements

Surface tension measurements were carried out by the use of a Krüss K12 tensiometer (Hamburg, Germany). The surface tension was determined with the single-measurement method using deionized water for instrument calibration (surface tension: 72.8 mN/m at 25.0 ± 0.1 °C). The platinum plate used in the measurements was cleaned with ultra-pure water (Millipore) followed by heating through flame. The surface tension of each solution was measured by successive addition of different aliquots of a stock solution to ultra-pure water after careful mixing and equilibration. All measurements were repeated at least two times.

In the case of FA-PILs/TPGS binary systems, stock solutions of the surfactants at the same molar concentration (20 mM, 5 mM, 1 mM, and 0.728 mM for C_7_CO-HTMG, C_9_CO-HTMG, C_9_CO-HTMG, and C_13_CO-HTMG, respectively) were prepared and subsequently mixed in order to maintain a fixed amount of FA-PIL with varying that of vitamin E TPGS.

The values of the critical micellar concentration (*cmc*) and the surface tension at the *cmc* (γ*_cmc_*) were determined from the intersection of the two straight lines obtained by surface tension curves in low- and high-concentration regions (γ-log C curves) using a linear regression analysis method.

Surface tension measurements were carried out under salt-free conditions.

### 2.3. Preparation and Physico-Chemical Characterization of Self-Assembling Micelles

Self-assembling micelles containing FA-PILs (C_n_PILs/Nano and C_n_PILs-IMQ/Nano) were prepared by introducing the selected FA-PIL (and IMQ, if any) in water, stirring overnight.

In the case of mixed self-assembling micelles (C_n_PILs-TPGS/Nano and C_n_PILs-TPGS-IMQ/Nano), the selected FA-PIL and vitamin E TPGS were heated over the melting point of both surfactants (50 °C); then, drug was added under continuous stirring to obtain a homogeneous blend. As a last step, water at the same temperature as the blend was added and the mixture was stirred overnight.

In all cases, the final mixture containing IMQ was filtered through cellulose acetate filters (0.22 m pore size, Minisart^®^ NML Syringe filters, Sartorius, Florence, Italy) to remove unloaded drug, aggregates, and foreign particulates.

The prepared micelles were characterized with respect to size, size distribution, and encapsulation efficiency (drug loading and entrapment). Size and size distribution of the micelles were determined by using a Dynamic Light Scattering (DLS) Beckman Coulter^®^ N4 Plus (Beckman Coulter s.r.l, Milan, Italy) following the procedure reported in Terreni et al. [14].

The average size for each formulation was determined on 6 runs of three different samples at two angles (62.6° and 90°) and run time of 200 s at 20 °C.

The entrapment efficiency, defined as the percentage of drug loaded inside the micelles with respect to the one formerly added to the formulation, was determined by high performance liquid chromatography (HPLC). To ensure complete destruction of the micelles and release of the incorporated drug, an aliquot of each formulation was diluted with methanol and the amount of drug was determined by HPLC analysis.

The entrapment and loading efficiency of IMQ were calculated using the following equations:

Entrapment efficiency, EE% = (mass of drug in micelles) × 100/(mass of drug added in the formulation)(1)

Loading efficacy, LE% = (mass of drug in micelles × 100)/(mass of drug added + mass of surfactants in the formulation)(2)

The pH sensitivity of the nanostructures was investigated by testing the distribution of the particle size in both the simulated endo/lysosome conditions (pH = 5.5) and physiological pH (pH = 7.4) at fixed time intervals (0 min, 30 min, 1, 4, and 24 h).

### 2.4. In Vitro Release of IMQ from Polymeric Micelles

In order to simulate the tumoral and endosomal environment, the in vitro release profiles of IMQ from the prepared micelles has been examined at pH 7.4 (physiological pH) and pH 5.5 (endosomal pH) using a dynamic dialysis method.

Briefly, the obtained micellar systems were dispersed in 1 mL of different buffer solution pH 7.4 (phosphate buffer saline, PBS) and pH 5.5 (sodium acetate buffer) and placed in a dialysis bag (MCWO 3500 Da, SpectraPore 3 Dialysis Membranes, Spectrum Labs, Breda, The Nederlands). The receptor medium consisted of 5 mL of buffer at the same pH maintained at 37 °C (body temperature) in a water bath with gentle stirring (20 rpm). At predetermined time intervals, 1 mL aliquots were withdrawn and replaced with an equal volume of fresh medium. All the experiments lasted 24 h and were performed in triplicate. The amount of drug in the samples was determined by HPLC.

### 2.5. In Vitro Cytotoxicity Analysis

The cytotoxicity on 501Mel melanoma cells of IMQ alone and that of IMQ loaded on non-cytotoxic concentrations of C_7_/C_13_CO-HTMG-TPGS/Nano were compared. 501Mel cells were kindly provided by Dr. Poliseno (Oncogenomics Unit, Core Research Laboratory, Istituto Toscano Tumori c/o IFC-CNR, Pisa, Italy).

IMQ was dissolved in DMSO and the C_7_/C_13_CO-HTMG-TPGS-IMQ/Nano alone and loaded with IMQ were dissolved in water.

Culture conditions and cell viability assay (3-(4,5-dimethylthiazol-2-yl)-5-(3-carboxymethoxyphenyl)-2-(4-sulfophenyl)-2H-tetrazolium; MTS) were performed in agreement with the ones reported in Carpi et al. [65]. After overnight attachment, cells were incubated with IMQ and C_7_/C_13_CO-HTMG-TPGS-IMQ/Nano at two different concentrations for 24 h. At the end of the treatment, 20 μL of MTS solution was added to each well and absorbance was read at 490 nm using the Infinite M200 NanoQuant instrument (Tecan, Salzburg, Austria). Optical density values from vehicle-treated cells were considered as 100% cell viability. For each treatment, the corresponding vehicle-treated cells were used as control, i.e., the treatment with DMSO alone at the corresponding concentration of IMQ represents the control of IMQ and cell medium represents the control of C_7_/C_13_CO-HTMG-TPGS-IMQ/Nano.

### 2.6. In Vitro Cutaneous Permeation and Distribution Studies

Porcine ears skin, used as a model, was obtained from freshly sacrificed animals in a local slaughterhouse as previously described in Tampucci et al. [17]. The pig hairs were abscised and the skin (about 1.46 ± 0.06 mm in thickness) was gently washed and placed in the Gummer-type diffusion cells, as in Reference [66]. The donor phase consisted of 200 microliters of the nanostructured formulation under study or Imunocare^®^ commercial product (Difa Cooper S.p.A., Caronno Pertusella (VA), Italy), cream containing 5% IMQ, used as a reference. The receiving phase consisted of either PBS pH 7.4 added with 1% serum albumin to enhance drug solubility or sodium acetate buffer pH 5.5 (not physiological pH), maintained at 37 °C and stirred at 600 rpm. At predetermined time intervals, the receiving phase (5 mL) was withdrawn for HPLC analysis and replaced with the same volume of fresh fluid, and sink conditions were maintained throughout the entire study. All experiments lasted 24 h and were replicated four times.

At the end of the permeation experiments, the skin was collected and treated following the procedure reported and validated in Monti et al. [66]. Briefly, in order to extract the drug, the minced skin was treated with 2.0 mL of 2% sodium lauryl sulphate (SDS, Sigma-Aldrich, Milan Italy) for 24 h at 37 °C, followed by a treatment with a methanol:chloroform (2:1) solution (3 mL) for 1 h at 37 °C under stirring. Finally, the mixture was centrifuged at 4000 rpm for 15 min and two hundred microliters of supernatant were dried in vacuo and subsequently dissolved in methanol for HPLC analysis. The percentage of recovery was in the range 88–96%.

### 2.7. Storage Stability of IMQ-Loaded FA-PILs-TPGS Mixed Nanostructures

IMQ-Loaded FA-PILs-TPGS mixed nanostructures were subjected to the freeze-drying process and the freeze-dried samples were stored in a desiccator for up to 30 days. The freeze-drying process was carried out in a Virtis apparatus (VirTis Wizard 2.0, SP Scientific, Warminster, PA, USA) as previously reported in Burgalassi et al. [67].

The freeze-dried samples, reconstituted with the original volume of water to restore the initial formulation, were subjected over time (15 and 30 days) to dimensional and HPLC analysis, to highlight, respectively, any changes in particle size and in drug loading with respect to the same formulation before freeze-drying. The experiments were performed in triplicate.

### 2.8. HPLC Analysis

A reverse-phase-HPLC method was used to determine the IMQ concentration in the samples with the apparatus previously described in Tampucci et al. [68]. The injection valve was a Rheodyne with a capacity of 20 μL. A reverse-phase C18 column (Kinetex C18 2.6 μm, 100 Å, 75 × 4.6 mm, Phenomenex, Torrance, CA, USA) connected to a KJ0-4282 safe guard (Phenomenex^®^, Italy) was used. Isocratic elution was performed using a mobile phase consisting of a mixture of CH_3_OH/CH_3_CN/H_2_O/TEA (180/270/530/20) filtered through a 0.45 μm pore size membrane filter. The detection wavelength was 242 nm, the flux was 0.5 mL/min, and the retention time was 6 min.

The amount of IMQ in the samples was determined by comparison with appropriate external standard curves obtained applying the least square linear regression analysis. For in vitro studies, the calibration curves were obtained by dissolving the drug in methanol and then diluting with the appropriate medium (buffer solutions at pH 5.5 or 7.4). In case of skin samples, the standard curves were obtained by adding increasing amounts of the drug to a blank biological matrix, as previously described [68].

### 2.9. Data Analysis

Data related to in vitro cytotoxicity analysis were reported as mean ± standard deviation (SD) of at least three independent experiments, each performed in triplicate. Statistical analysis was performed using GraphPad Prism software, version 8.0 (GraphPad Software Inc., San Diego, CA, USA) using the t-test. A *p*-value < 0.05 was considered statistically significant.

For the in vitro permeation/penetration study, linear regression analysis of pseudo steady-state diffusion plots allowed calculation of the following parameters: steady-state flux (J), as Q/At, in which Q is the amount of drug diffusing across the area A in the time t; lag time (t_L_), as the time needed by the drug to saturate the membrane and to reach the receiving phase, estimated from the X-intercept of the regression line, and drug cumulative amount permeated at end of the 24 h (Q_24h_). Furthermore, the drug content (Q_skin_) retained in the skin at the end of the experiment was calculated following the extraction procedure. A retention factor (RF), calculated as Q_skin_/C_v_, is reported to compare the amount of drug retained in the skin after application of each formulation, taking into account the initial concentration of the drug (C_v_).

The significance of the differences between permeation parameters was assessed by GraphPad Prism software. Group comparison was assessed by using the Student’s two-tailed unpaired t-test (*p* < 0.05).

## 3. Results and Discussion

The aim of the present work was to demonstrate the ability of a new set of FA-PILs (Figure 1) to behave as surfactants and originate micelles with nanometric dimensions. Ultimately, these materials were intended to offer a distinct advantage to develop a nanostructured pH-triggered drug delivery system for application in the skin cancer therapy. In addition, the effect of selected FA-PILs in the formation of mixed micelles containing a nonionic surfactant, vitamin E TPGS, was evaluated, as mixed micellar systems are known to improve thermodynamic stability (lower CMC) and kinetics, to favor drug encapsulation and a more accurate control of dimensions [69].

### 3.1. Preparation and Characterization of Fatty Acid-Protic Ionic Liquids (FA-PILs)

#### 3.1.1. NMR Analysis

The FA-PILs structures were assessed by ^1^H and ^13^C-NMR analyses (Supplementary Figure S1–S4). The high purity of FA-PILs was ascertained by integration of the peaks in the ^1^H-NMR spectra. In particular, the comparison of the integral values of the methyl groups attached to the guanidinium nitrogen atoms (2.89–2.95 ppm) and of the methylene group adjacent to the carboxylate group (2.07–2.16 ppm) confirmed the 1:1 ratio between cation and anion.

#### 3.1.2. Differential Scanning Calorimetry (DSC)

**Figure 2:**
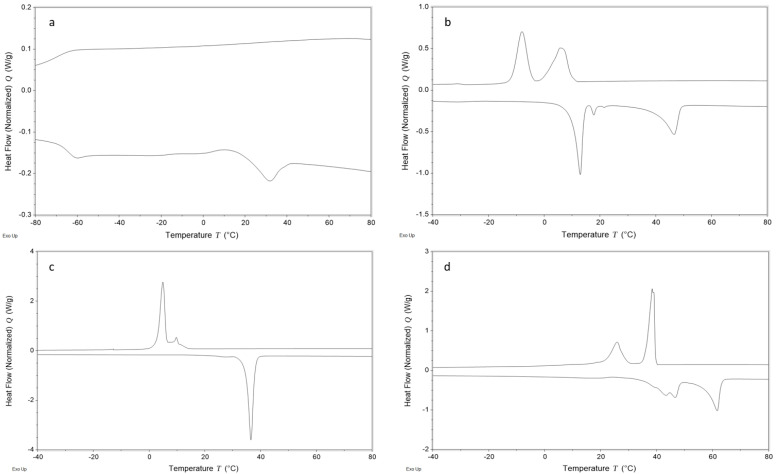
Differential scanning calorimetry (DSC) of C_7_CO-HTMG (**a**), C_9_CO-HTMG (**b**), C_11_CO-HTMG (**c**), and C_13_CO-HTMG (**d**), at a scanning rate of 5 °C/min.

The thermal behavior of FA-PILs was investigated by differential scanning calorimetry (DSC) at different scanning rates (2, 5, and 10 °C/min) in the temperature range going from −90 to 100 °C under a nitrogen flow. Three general thermal behaviors have been found for ILs and are classified as: type I behavior for ILs which have little tendency to crystallize and show only glass transitions, type II for those ILs which form crystals on cooling and display the related melting event during the heating, thus acting as low melting salts, and type III when cold crystallization and melting are both recorded during the heating half cycle [70,71]. For FA-PILs, more complicated thermographs were observed which could be attributable to the presence of polymorphs (Supplementary Figure S5–S8). The presence of several thermal events is quite common for fatty acids ILs, especially for those constituted by long-chain fatty acid anions [41,72]. As expected, C_7_CO-HTMG, which features the shortest alkyl chain of the series, exhibits a type I thermal behavior at 2 °C/min that shifts towards the type III behavior at higher scanning rates (5 and 10 °C/min), without the presence of polymorphic events (Figure 2a). C_9_CO-HTMG, C_11_CO-HTMG, and C_13_CO-HTMG showed a polymorphic behavior with two main transitions both in heating and cooling ramps. Despite their polymorphic character, these ILs show a type II thermal behavior with the melting step in the heating ramp and the crystallization in the cooling ramp (Figure 2b–d), with the exception of C_13_CO-HTMG, which displays a type III behavior at 10 °C/min. All the phase transitions found for FA-PILs are summarized in Table 1.

#### 3.1.3. Surface Tension Measurements

**Figure 3:**
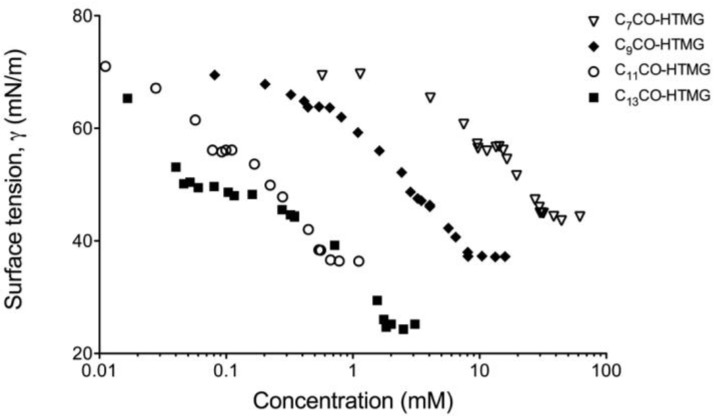
Surface tension (γ) isotherms as a function of FA-PILs concentration in aqueous solution at 25 °C.

The surface tension of FA-PILs was measured to evaluate their surface activity. Figure 3 shows the surface tension (γ) versus concentration (C) plot obtained for the aqueous solutions of the surface-active FA-PILs.

It is apparent that the surface tension of each FA-PIL gradually decreases by increasing the IL concentration, first, to reach a nearly constant value (γ_*cmc*1_) represented by a plateau region in the surface tension isotherms, followed by a second plateau (γ_*cmc*2_) indicating a second *cmc* value. The values of *cmc*_1_ and *cmc*_2_ for each FA-PILs are shown in Table 2. An effect of the length of the hydrocarbon chain on the *cmc*s, which decrease as the alkyl chain increases, can be observed, confirming previously reported data for similar structures [73].

The existence of two *cmc*s is not surprising for micellar systems or for ionic liquids in aqueous solution. Dimensional growth of the micelles is reported to take place in two well-defined stages.

Generally, the micelles have a spherical shape at surfactant concentrations higher than *cmc*. With a further increase in concentration, the micelles tend to elongate to form rod-like micelles, which can further grow to a relatively high surfactant concentration to form worm-like micelles. Therefore, at surfactant concentrations higher than the first *cmc* (*cmc*_1_), aggregation of the micelles in more complex structures may occur. Each added surfactant molecule aggregates into small micelles, until the second *cmc* (*cmc*_2_) is reached; after that, the further added amphiphiles begin to organize themselves into larger non-surface-active linear micelles [74,75].

Finally, it is important to highlight the absence of a minimum in the breakpoint region that generally occurs in presence of highly surface-active impurities. This is further pointed out by sharp endothermic peaks obtained in the DSC experiments, which confirms the purity of the synthesized FA-PILs [76,77].

**Figure 4:**
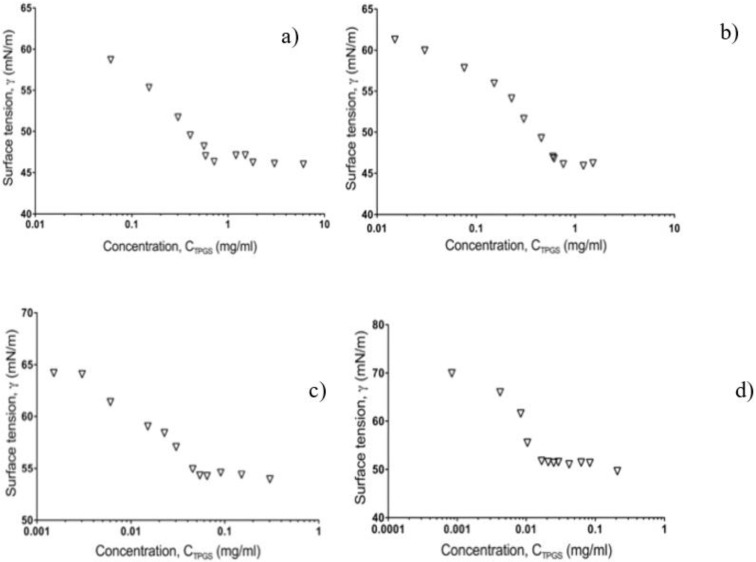
Surface tension isotherms of mixed systems (**a**) C_7_CO-HTMG/TPGS, (**b**) C_9_CO-HTMG/TPGS, (**c**) C_11_CO-HTMG/TPGS, and (**d**) C_13_CO-HTMG/TPGS at different molar ratio.

Mixing of surfactants with ILs endowed with surfactant properties can considerably alter the physico-chemical properties of the system [78]. In the present work, the effect of newly synthesized FA-PILs on the micellar behavior of a nonionic surfactant such as vitamin E TPGS was investigated. The plots related to the surface tension isotherms of the binary mixtures FA-PIL/TPGS are reported in Figure 4. The measured *cmc* of vitamin E TPGS (0.23 mg/mL) was found in good agreement with the literature value for the pure amphiphile (0.02% *w/w*).

It is known that micelles formation is governed both by electrostatic interactions of the polar head of the surfactants and by the hydrophobic interactions of the hydrocarbon chains [78]. When the vitamin E TPGS was mixed with FA-PILs based on short-chain fatty acids, namely caprylic and capric acids, the *cmc* values for vitamin E TPGS in the binary mixtures (C_7_CO-HTMG/TPGS and C_9_CO-HTMG/TPGS) were higher than that of the vitamin alone (0.58 and 0.45 mg/mL, respectively). Conversely, when FA-PILs based on longer chain fatty acids, such as lauric and myristic acids (C_11_CO-HTMG and C_13_CO-HTMG), were employed in the binary mixtures, the *cmc* of vitamin E TPGS was lower than that of the single component (0.043 and 0.014 mg/mL), probably due to an increase in hydrophobic interactions. Indeed, vitamin E TPGS-, the C12-, and the C14 fatty acids-based ionic liquids all possess a distinct hydrophobic character, which can favor the micellization process.

### 3.2. Preparation and Characterization of Self-Assembling Nanostructures

All the synthesized FA-PILs were screened for their capability to originate micelles when dispersed in water at a concentration above their *cmc*_1_. A pool of formulations was tuned up and the composition of the mixtures along with their physico-chemical characterization in terms of hydrodynamic diameter (D_h_) at two different angles (62.6° and 90°) and polydispersity index (P.I.) is reported in Table 3.

In the case of C_9_CO-HTMG, C_11_CO-HTMG, and C_13_CO-HTMG, micelles of dimensions around 200 nm and with a uniform size distribution at the two angles tested were obtained only when the concentration of ionic liquid was between *cmc*_1_ and *cmc*_2_. Conversely, in the case of C_7_CO-HTMG, a population of nano-sized micelles was formed only above the *cmc*_2_, probably due to the IL’s shorter alkyl chain. In any case, the dimensional distribution obtained with the formulation C_7_CO-HTMG/Nano showed a high polydispersity index (P.I. = 0.709) indicative of a not homogeneous size. Furthermore, it was impossible to detect the formation of a colloidal structure at the 62.6° angle.

On the basis of the results obtained with both the preliminary formulation study and the surface tension analysis, mixed systems containing FA-PILs and vitamin E TPGS were prepared with the aim of decreasing the dimension of the micelles and exploiting the capacity of vitamin E TPGS to inhibit the MDR. The prepared formulations are reported in Table 3 and it is evident that the mixture with the nonionic surfactant vitamin E TPGS led to the formation of micelles with lower dimension (D_h_ in the range from 7.67 to 42.37 nm) with respect to the system containing only the FA-PIL. In particular, it is interesting to highlight that it was possible to use the FA-PIL under its *cmc*_2_ by mixing C_7_CO-HTMG and vitamin E TPGS (C_7_CO-HTMG-TPGS/Nano). By adopting this strategy, micelles with a low D_h_ and a narrow dimensional distribution at the two angles tested could be obtained, which is indicative of a spherical shape of the colloidal system. Conversely, when C_11_CO-HTMG was used in the binary system, a not homogeneous dispersion was obtained (P.I. between 1.163 and 1.322), probably due to both the FA-PIL concentration near the *cmc*_2_ and the high molar ratio between the two ionic and non-ionic surfactants (1:10 molar ratio).

**Scheme 2:**
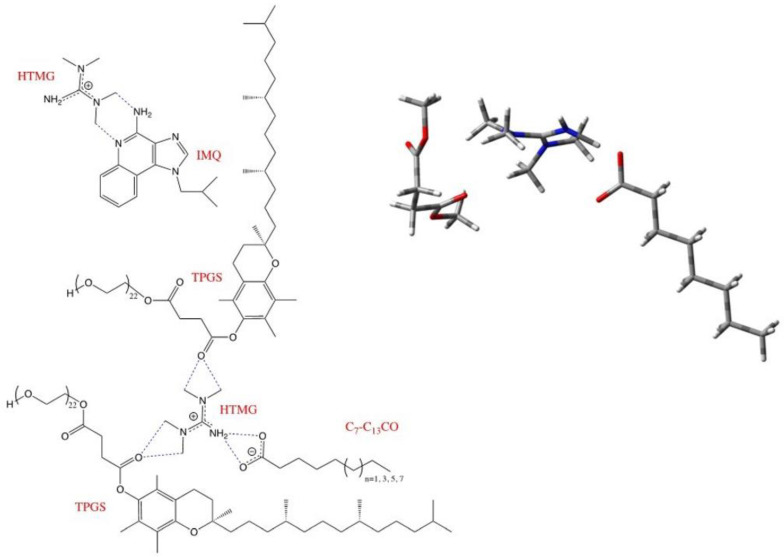
Left: Scheme of the possible molecular interactions in C_7_-C_13_CO-HTMG-TPGS/Nano and HTMG-IMQ/Nano (potential hydrogen bonds are highlighted in blue). Right: DFT optimized structure of a 1:1:1 cluster of C_7_CO-HTMG-Model of TPGS. In the model, TPGS has been reduced to a dimethyl succinic ester. This model contains all the functional g11roups needed for HTMG–TPGS interactions. From the geometry, the interactions of one carboxylic group with the HTMG methyl groups is evident. The cluster presents a stability of 59.50 KJ/mol with respect to the constituents. More details and larger scale picture are provided in the Supplementary Materials, Figure S9.

Finally, a ternary system designed with our best results in mind has been formulated by mixing C_7_CO-HTMG and C_13_CO-HTMG with vitamin E TPGS. C_7_/C_13_CO-HTMG-TPGS/Nano showed the best characteristics in terms of both D_h_ and P.I. (10.31 nm and 0.052 respectively, at a 90° angle). Regarding the structure of the above-mentioned micelles, a spherical shape could be hypothesized (10.1 and 10.3 nm at 62.6° and 90° angles). Besides, as HTMG presents a positive charge and a triangular shape, it can act as a hydrogen bond fitting using acidic hydrogen on NH_2_ and CH_3_ groups. A reasonable hydrogen-bonded cluster is represented in Scheme 2. Therefore, HTMG could act as a sort of crosslinker strengthening the micellar structure.

C_7_/C_13_CO-HTMG-TPGS/Nano have been selected as a delivery system of IMQ. The topical delivery of IMQ is extremely challenging due to its very low aqueous solubility in water medium (0.01 mM) and poor skin permeation capacity, despite its low molecular weight (MW 240.30 g/mol) and a logP of 2.6 [79]. Therefore, IMQ can be considered a good candidate to be formulated in a nanostructured drug delivery system with the aim of improving its penetration and cytotoxicity at a well-tolerated dosage.

When IMQ was formulated in the micellar system based on C_13_CO-HTMG, alone or in combination with vitamin E TPGS (C_13_CO-HTMG-IMQ/Nano and C_13_CO-HTMG-TPGS-IMQ/Nano, respectively), the obtained micelles presented a D_h_ in the range 206–269 nm (Table 4).

It should be noticed that there were no differences in D_h_ between empty and IMQ-loaded micelles based only on C_13_CO-HTMG (264.7 and 226.4 nm vs. 251.7 and 206.5 nm respectively, for C_13_CO-HTMG/Nano and C_13_CO-HTMG-IMQ/Nano); on the other hand, the hydrodynamic diameter of the C_13_CO-HTMG-TPGS-IMQ/Nano markedly increased due to IMQ encapsulation (269.5 and 218.7 nm vs. 15.5 and 14.0 nm for loaded and empty micelles).

Conversely, when the ternary system containing IMQ was formulated (C_7_C_13_CO-HTMG-TPGS-IMQ/Nano), only a slight increase in both the D_h_ and P.I. was noticed with respect to the empty micelles, maybe because of the lower entrapment efficiency (EE%) of C_7_C_13_CO-HTMG-TPGS-IMQ/Nano with respect to C_13_CO-HTMG-TPGS-IMQ/Nano.

Regardless, it is worth highlighting that in all cases, notwithstanding the low EE%, the drug solubility was enhanced from 3.6-fold to 83-fold (with C_7_C_13_CO-HTMG-TPGS-IMQ/Nano and C_13_CO-HTMG-TPGS-IMQ/Nano, respectively) with respect to the value reported in the literature [79], indicating the suitability of the proposed nanostructures as a drug delivery system for IMQ.

Regarding the structure of the proposed nanostructures, HTMG could form hydrogen bonds with imiquimod, too, as represented in Scheme 2 for C_7_C_13_CO-HTMG-TPGS-IMQ/Nano. IMQ could be, in this way, incorporated in the micellar structure.

### 3.3. pH-Dependent Release of IMQ from the Nanostructures

**Figure 5:**
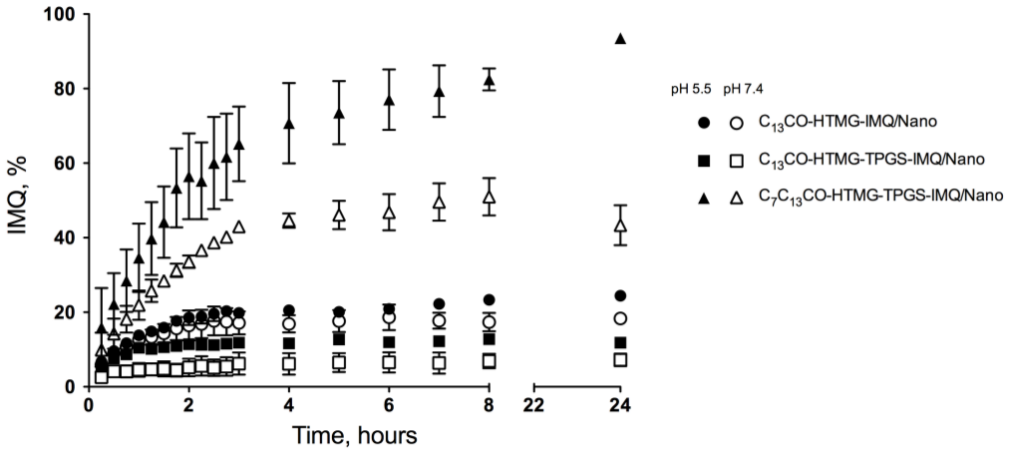
In vitro pH-dependent release profiles of IMQ from the selected micelles at pH 5.5 and pH 7.4 (mean ± standard deviation (SD), *n* = 3).

The release of IMQ from C_n_PILs-IMQ/Nano and C_n_PILs-TPGS-IMQ/Nano was performed at 37 °C for 24 h in different buffer solutions, pH 5.5 and pH 7.4, representing physiological pH and endosomal pH, respectively. As shown in Figure 5, all the selected micelles exhibited pH-triggered release behavior: the release of drug was inversely proportional to the pH value. Disappointingly, C_13_CO-HTMG-IMQ/Nano and C_13_CO-HTMG-TPGS-IMQ/Nano were able to release only a small amount of drug during the 24 h. On the other hand, no more than 50% of the total IMQ was released from C_7_C_13_CO-HTMG-TPGS-IMQ/Nano after 24 h at pH 7.4, indicating that the micelles were stable under physiological conditions and did not undergo specific drug release before reaching the tumor site. Furthermore, at pH 5.5, a faster release of IMQ from C_7_C_13_CO-HTMG-TPGS-IMQ/Nano was observed, reaching 94% after 24 h and suggesting pH-sensitive drug release. The results obtained demonstrated that the proposed nanostructures possess a tunable, pH-dependent ability to affect the IMQ release. A selective drug release at acidic pH would be desirable in the skin cancer therapy, since the reported pH of healthy melanocytes is about 7.4, while the melanoma microenvironment exhibits a 5.5–6.5 pH due to lactic acid accumulation [80]. The designed FA-PILs take advantage of this phenomenon. As anticipated, the partial protonation of the fatty acid carboxylates of the FA-PILs in the acidic environment likely leads to a loss of the electrostatic interactions with the HMTG cationic headgroup and ultimately to the destabilization of the self-assembled systems. This effect becomes especially evident when the shortest carboxylate (C_7_CO) is employed as part of the FA-PIL, possibly on account of the weaker hydrophobic interactions, which in turn facilitate the collapse of the micelle.

**Figure 6:**
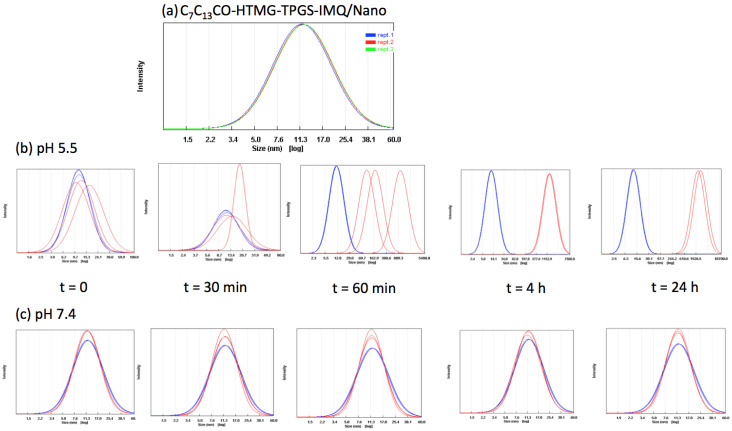
Particle size distribution of C_7_C_13_CO-HTMG-TPGS-IMQ/Nano: (**a**) in deionized water, (**b**) in the simulated endo/lysosome conditions (pH = 5.5, red line) vs. deionized water (blue line), and (**c**) at physiological pH (pH = 7.4, red line) vs. deionized water (blue line) at predetermined time intervals.

In support of the above assertions, the pH-sensitive behavior of C_7_C_13_CO-HTMG-TPGS-IMQ/Nano was further investigated to demonstrate the structural changes of these hybrid micelles in an acidic environment. The concerned micelles were incubated in a pH 5.5 buffer solution simulating the tumor acidic/endosomal environment and the D_h_ and size distribution were determined at pre-determined time intervals, comparing to those measured at physiological pH. At the zero time point, the micelles were in a narrow size range with low P.I.; subsequently, after the exposure to acidic conditions, both the particles’ size and the percentage of larger particles gradually increased (Figure 6a), while they remained unaffected under normal physiological conditions (Figure 6b). These results confirm the pH-sensitivity of the C_7_C_13_CO-HTMG-TPGS-IMQ nanomicelles, suggesting that the acidic environment of cancer tissues can trigger their swelling and collapse with consequent rapid release of IMQ, enhancing the drug cytotoxic potential [20].

### 3.4. In Vitro Cell Viability

Imiquimod is an anticancer agent showing both immune-stimulation activity and a direct cytotoxic/anti-proliferative action [65,81,82,83]. Therefore, in vitro melanoma cells have been used in the present study as the simplest cancer model to verify the usefulness of the proposed nanostructured system. A cell viability assay was performed on 501Mel melanoma cells to determine the effectiveness of the IMQ-containing micelles in inhibiting cell growth. A preliminary evaluation on the empty micelles (C_7_/C_13_CO-HTMG-TPGS/Nano) showed that they did not induce a significant effect on cells’ viability after 24 h of incubation up to a concentration limit of each component related to their amount in the formulation: C_7_CO-HTMG 86.6 g/mL, C_13_CO-HTMG 3.33 g/mL, and vitamin E TPGS 504 g/mL. The C_7_/C_13_CO-HTMG-TPGS-IMQ/Nano were tested in the same conditions and the IMQ concentration in the well was 200 ng/mL. The effect of this concentration and its 1/10 were compared to the one of the IMQ alone.

**Figure 7:**
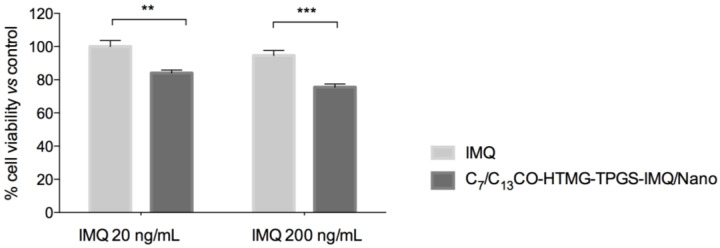
Cytotoxicity of free IMQ and C_7_/C_13_CO-HTMG-TPGS-IMQ/Nano on 501Mel cells. 501Mel cells (4 × 10^3^ cells) were seeded in 96-well plates and, after overnight attachment, treated with different concentrations of IMQ or IMQ-loaded C_7_/C_13_CO-HTMG-TPGS/Nano for a further 24 h. Cell viability was determined by the MTS assay. Data are presented as means ± SD of three independent experiments, each performed in triplicate. ** *p* < 0.01; *** *p* < 0.001.

As shown in Figure 7, a significant decrease of cell viability was evident with both the IMQ concentrations evaluated (about 15% and 25% of decrease for 20 and 200 ng/mL of IMQ, respectively) when formulated into the nanostructures, but not when tested alone. This latter evidence agrees with previous reports where IMQ on the same cells [65] and in other cancer cells [83,84] was cytotoxic at concentrations greater than 1 μg/mL. This suggests an improved efficacy of IMQ when formulated in the nanomicellar system.

### 3.5. In Vitro Cutaneous Permeation and Distribution Studies of the Selected Formulation

The behavior of the formulation C_7_C_13_CO-HTMG-TPGS-IMQ/Nano was further investigated in a set of in vitro permeation studies using pig ear skin as a model. These experiments were carried out to evaluate the ability of the micellar formulation to deliver the drug selectively into the skin, where the target site for the topical treatment of melanoma is located, while reducing systemic absorption to a minimum.

The results of the in vitro experiments are summarized in Table 5, where the relevant permeation parameters (flux, lag time, amount of drug permeated and retained in the skin after 24 h) are reported. The commercial product Imunocare^®^ was taken as a term of comparison.

It is possible to observe that both tested formulations produced a transcutaneous permeation of drug dependent on the pH of the receiving phase; in particular, Imunocare^®^ and C_7_C_13_CO-HTMG-TPGS-IMQ/Nano tended to produce a significantly greater permeation at pH 5.5 with respect to physiological pH. Any significant difference in the permeation parameters was found between the two formulations tested at each pH. The pH-sensitive behavior of the self-assembling mixed micelles could be responsible for the higher amount of drug permeated in acidic conditions, as well as the dimensions of the nanostructure having a D_h_ 20 nm could favor the permeation of IMQ through intact skin, confirming data in Reference [60]. On the other hand, the commercial formulation contained a high amount of surfactants and isostearic acid with a solubilizing role, facilitating imiquimod permeation as demonstrated by Telò et al. [16].

One of the greatest challenges in topical application of anticancer drugs is to obtain a drug amount in the skin sufficient to give a therapeutical effect. Nanocarriers could improve drug targeting due to their ability of addressing the tumor cells, contemporaneously markedly reducing the amount of drug applied on the skin, and avoiding the direct contact of the drug with the cutaneous surface [10].

**Figure 8:**
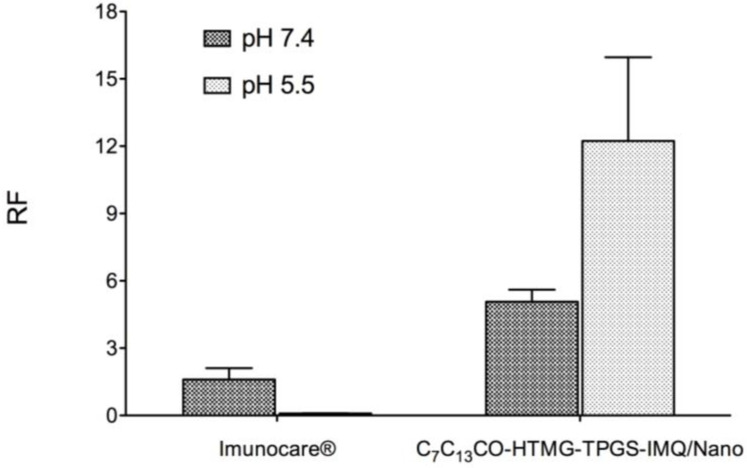
Retention factor (RF) calculated as the amount of drug retained in the skin after application of each formulation with respect to the initial concentration of the drug.

An important issue for a possible application on tumor tissue is that the micellar formulation C_7_C_13_CO-HTMG-TPGS-IMQ/Nano produced a significantly higher amount of IMQ retained in the skin at the acidic tumor pH with respect to that obtained at physiological pH, while for the commercial formulation, any difference in the behavior at the two pHs was detected. The commercial formulation produced a 26.8-fold (pH 7.4) and 7.3-fold (pH 5.5) higher amount of drug retained in the skin with respect to C_7_C_13_CO-HTMG-TPGS-IMQ/Nano. However, it is worth mentioning that when the micellar formulation was applied on the skin, the amount of IMQ administrated was about 1300-fold lower with respect to Imunocare^®^ (7.6 g vs. 10,000 g, respectively), suggesting that the nanostructured formulation was able to improve drug partitioning through the skin, as evident from the histograms in Figure 8, where the retention factor (RF) is reported to compare the amount of drug retained in the skin after application of each formulation, taking into account the initial concentration of the drug.

Besides, it should be noted that the commercial formulation is a semisolid formulation, while C_7_C_13_CO-HTMG-TPGS-IMQ/Nano is a colloidal dispersion in liquid form, which in the future will need to be formulated with a rheological agent in order to be applied on the skin surface favoring an intimate contact of the drug with the skin. Anyway, the amount of IMQ (5.93 ± 0.64 g/mL) retained in the skin at pH 5.5 with the micellar formulation was definitely higher than both the 200 ng/mL found to be effective in the in vitro cell viability assay performed on 501Mel melanoma cells (paragraph 3.6) and the IC_50_ value reported in literature (2 M) [85]. Moreover, the lower amount of drug that comes into contact with the skin could reduce irritation phenomena common in long-term skin cancer topical therapy.

### 3.6. Storage Stability of the IMQ-Loaded FA-PILs-TPGS Mixed Nanostructures Selected

The freeze-drying process has been extensively investigated to stabilize a variety of nanocarriers, but only a few reports deal with lyophilization of micellar systems [86]. The C_7_C_13_CO-HTMG-TPGS-IMQ/Nano formulation was subjected to the lyophilization process in order to stabilize the nanostructured system during storage for subsequent redispersion in water at the time of use. The freeze-dried sample obtained showed a soft appearance and was readily redispersible with deionized water to form a clear and colorless solution. The results of the dimensional analysis on reconstituted lyophilizates are shown in Table 6. After redispersion with water of the freshly prepared lyophilizate, it was possible to obtain micelles with dimensions comparable to the original ones and with uniform distribution. The dimensional analysis after 15 and 30 days of storage of the samples, reconstituted immediately before analysis and filtered through 0.22 µm filters, did not show an increase in either the particle size or the P.I., indicating the absence of aggregation phenomena during storage. Moreover, the amount of IMQ entrapped into the micelles remained stable during the observation period (95.30% and 97.99% of the initial amount after 15 and 30 days, respectively). The observed change from the initial content of drug remained lower than 5%, meeting the regulatory acceptance criteria for a finished pharmaceutical product, as described in the Annex 2 of the World Health Organization (WHO) Technical Report Series, No. 953: Stability testing of active pharmaceutical ingredients and finished pharmaceutical products (2009). Therefore, the freeze-drying method could represent a successful way to preserve the nano-dispersed system from instability phenomena.

## 4. Conclusions

The design of new biodegradable, nontoxic, and pH-sensitive polymeric materials, suitable to be used as drug delivery systems, is a great challenge for the pharmaceutical community. Moreover, one of the objectives in the above-mentioned field is to improve the effectiveness in tumor targeting, by selectively addressing cancer tissues and sparing healthy ones. The combination of newly synthesized fatty acid-protic ionic liquids (FA-PILs) and vitamin E TPGS led to the formation of self-assembling micelles of nanometric size, able to encapsulate the anticancer drug IMQ and to release it in cancer tissues with a pH-triggered mechanism; besides, the proposed nanosystem is capable of producing effective drug amounts at the action site (i.e., the skin) following topical application, thus representing a potential drug delivery system for skin cancer treatment.

## Figures and Tables

**Table 1:** Glass transition (T_g_), crystallization (T_c_), cold crystallization (T_cc_), and melting (T_m_) temperatures for FA-PILs with HTMG cation.

Anion.	Scanning Rate (°C/min)	Temperature (°C)			
		T_g_	T_c_	T_cc_	T_m_
C_7_COO	2	−69.7	--	--	--
	5	−65.5	--	10.7	31.7
	10	−63.1	--	11.4	35.5
C_9_COO	2	--	−6.1	--	17.8
			−12.8		39.6
	5	--	5.8	--	12.9
			−8.1		46.7
	10	--	7.6	--	11.6
			−12.4		48.6
C_11_COO	2	--	13.6	--	36.0
	5	--	9.8	--	36.5
			4.9		
	10	--	8.4	--	33.2
			2.6		36.9
C_13_COO	2	--	37.9	--	47.6
			32.9		59.7
	5	--	38.4	--	46.7
			25.9		61.7
	10	--	35.9	26.4	45.7
			22.0		62.2

**Table 2:** Critical micelle concentrations (*cmc*) and surface tension at *cmc* (γ*_cmc_*) values of the FA-PILs under study.

FA-PIL	*cmc*1 mM	*cmc*2 mM	γ*_cmc_*_1_ mN/M	γ*_cmc_*_2_ mN/M
C_7_CO-HTMG	9.72	30.88	56.30	45.18
C_9_CO-HTMG	0.48	7.18	63.77	37.26
C_11_CO-HTMG	0.08	0.56	55.74	37.73
C_13_CO-HTMG	0.05	1.80	49.99	25.75

**Table 3:** Composition of the mixtures used for the preparation of CnCOPILs/Nano and CnCOPILs-TPGS/Nano along with the related physico-chemical parameters, hydrodynamic diameter (D_h_) at two different angles, 62.6° and 90°, and polydispersity index (P.I.); n.d.: not detectable.

Formulation CnCOPILs/Nano	FA-PIL		Concentration mM	D_h_ (nm)		P.I.	
				62.6°	90°	62.6°	90°
C_7_CO-HTMG/Nano	C_7_CO-HTMG		45.50	n.d.	205.1 ± 38.9	n.d.	0.709
C_9_CO-HTMG/Nano	C_9_CO-HTMG		3.688	253.5 ± 1.7	200.6 ± 12.4	0.233	0.065
C_11_CO-HTMG/Nano	C_11_CO-HTMG		0.100	236.9 ± 3.5	201.9 ± 19.9	0.218	0.273
C_13_CO-HTMG/Nano	C_13_CO-HTMG		0.728	264.7 ± 1.3	226.4 ± 9.31	0.124	0.158
**Formulation** **CnCOPILs-TPGS/Nano**	**FA-PIL** **mM**		**TPGS** **mM**	**D_h_** **(nm)**		**P.I.**	
				**62.6°**	**90°**	**62.6°**	**90°**
C_7_CO-HTMG-TPGS/Nano	25.00		25.00	13.6 ± 0.4	12.0 ± 0.8	0.331	0.112
C_9_CO-HTMG-TPGS Nano	5.00		50.00	8.6 ± 0.06	7.7 ± 0.3	0.440	0.351
C_11_CO-HTMG-TPGS Nano	0.40		4.00	42.4 ± 15.0	20.3 ± 0.7	1.322	1.163
C_13_CO-HTMG-TPGS/Nano	1.455		1.455	15.5 ± 1.8	14.0 ± 1.0	0.377	0.272
C_7_C_13_CO-HTMG-TPGS/Nano	25.0 *	1.455 ^§^	25.00	10.1 ± 0.1	10.3± 0.7	0.135	0.052

* related to C_7_CO-HTMG; ^§^ related to C_13_CO-HTMG.

**Table 4:** Technological characteristics: hydrodynamic diameter (D_h_), polydispersity index (P.I.), amount of IMQ solubilized (IMQ_sol_), and entrapment and loading efficiency (EE% and LE%).

Formulation	IMQ mM	D_h_ (nm)		P.I.		IMQ_sol_ mM	EE %*w/w*	LE %*w/w*
		62.6°	90°	62.6°	90°			
C_13_CO-HTMG-IMQ/Nano	5.00	251.7 ± 5.6	206.5 ± 16.9	0.245	0.270	0.036	0.72	0.60
C_13_CO-HTMG-TPGS-IMQ/Nano	5.00	269.5 ± 1.4	218.7 ± 1.0	0.250	0.683	0.83	16.60	5.10
C_7_C_13_CO-HTMG-TPGS -IMQ/Nano	5.00	13.6 ± 0.2	12.3 ± 0.3	0.364	0.229	0.18	3.60	0.25

**Table 5:** Permeation parameters: flux (J), lag time (t_L_), and amount of drug permeated through (Q_24h_) and retained into (Q_skin_) the skin at the end of the experiment (24 h) from the formulations under study (mean ± standard error SE, *n* = 3).

Formulation	pH Receiving Phase	J ng/cm^2^·h	t_L_ h	Q_24h_ g/cm^2^	Q_skin_ g/cm^3^
Imunocare^®^	7.4	7.97 ± 2.53	4.24 ± 0.10	0.16 ± 0.05	73.70 ± 22.81
	5.5	86.64 ± 8.21 *	4.03 ± 0.36	1.68 ± 0.13 *	43.56 ± 2.94
C_7_C_13_CO-HTMG-TPGS-IMQ/Nano	7.4	7.86 ± 0.19	4.72 ± 1.95	0.15 ± 0.01	2.75 ± 0.19
	5.5	107.40 ± 16.32 *	4.47 ± 0.40	2.11 ± 0.36 *	5.93 ± 0.64 *

* significantly different from the same parameter at pH 7.4.

**Table 6:** Stability evaluation of C_7_C_13_CO-HTMG-TPGS-IMQ/Nano before and after freeze-drying at different times of storage.

Time	D_h_ (nm)		P.I.		IMQ_sol_ mM
	62.6°	90°	62.6°	90°	
Before freeze-drying	13.1 ± 0.22	12.0 ± 0.24	0.47 ± 0.01	0.19 ± 0.04	0.149 ± 0.021
0	11.8 ± 0.08	12.0 ± 0.59	0.14 ± 0.01	0.08 ± 0.31	0.145 ± 0.020
15 days	10.2 ± 0.11	10.4 ± 0.80	0.13 ± 0.03	0.21 ± 0.31	0.142 ± 0.024
30 days	12.2 ± 0.14	12.2 ± 1.00	0.19 ± 0.03	0.03 ± 0.59	0.146 ± 0.022

## Supplementary Materials

The following are available online at https://www.mdpi.com/1999-4923/12/11/1078/s1, Figure S1: ^1^H NMR of C_7_CO-HTMG, Figure S2: ^1^H NMR of C_9_CO-HTMG, Figure S3: ^1^H NMR of C_11_CO-HTMG, Figure S4: ^1^H NMR of C_13_CO-HTMG, Figure S5: Differential scanning calorimetry (DSC) of compound C_7_CO-HTMG at different scanning rates, Figure S6: Differential scanning calorimetry (DSC) of compound C_9_CO-HTMG at different scanning rates, Figure S7: Differential scanning calorimetry (DSC) of compound C_11_CO-HTMG at different scanning rates, Figure S8: Differential scanning calorimetry (DSC) of compound C_13_O-HTMG at different scanning rates.
