# Supplementary material for: pH-Responsive Nanostructures Based on Surface Active Fatty Acid-Protic Ionic Liquids for Imiquimod Delivery in Skin Cancer Topical Therapy
Source: Pharmaceutics. 2020 Nov 11;12(11):1078. doi: 10.3390/pharmaceutics12111078 (PMC7697672; doi:10.3390/pharmaceutics12111078)
Supplement: Supplementary file 1 [file pharmaceutics-12-01078-s001.pdf]

pharmaceutics

MDPI

Supplementary Materials: pH-Responsive Nanostructures based on Surface Active Fatty Acid-Protic Ionic Liquids for Imiquimod Delivery in Skin Cancer Topical Therapy

Silvia Tampucci *, Lorenzo Guazzelli *, Susi Burgalassi, Sara Carpi, Patrizia Chetoni, Andrea Mezzetta, Paola Nieri, Beatrice Polini, Christian Silvio Pomelli, Eleonora Terreni and Daniela Monti

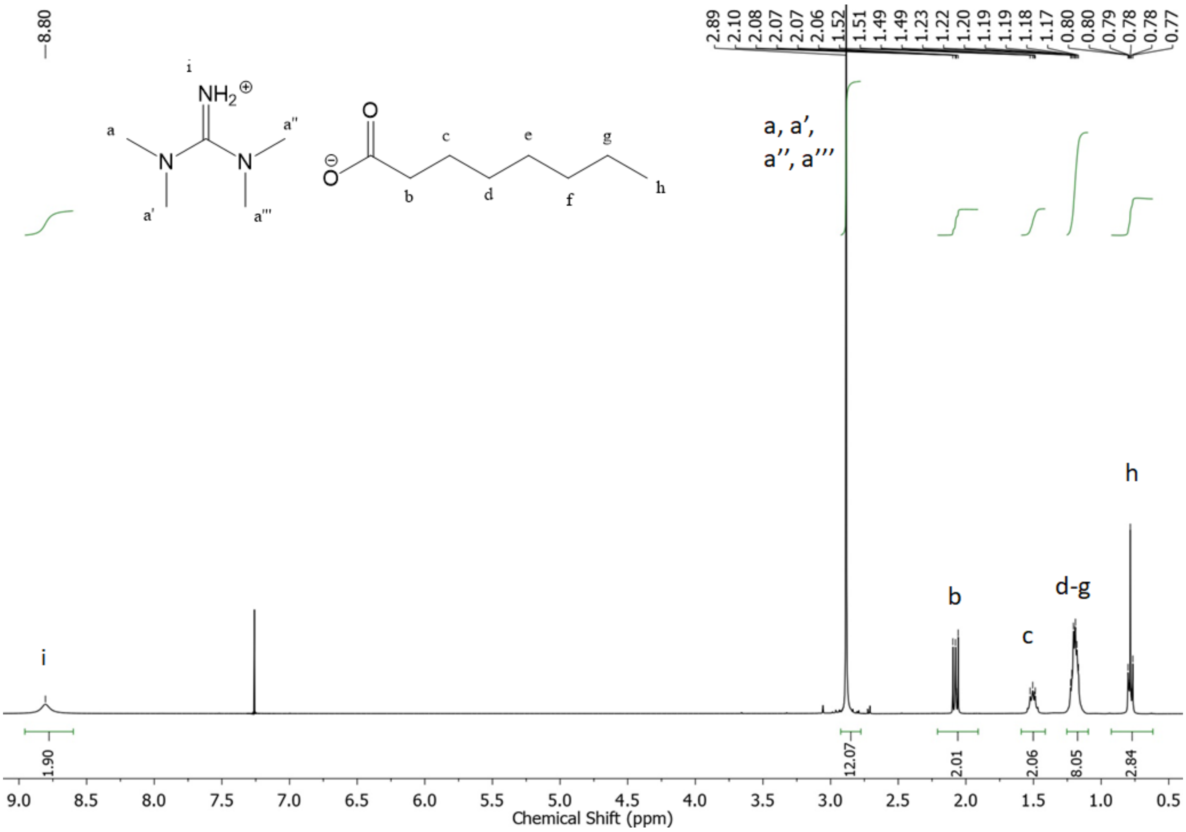

Figure S1. ^{1}H NMR of C_7CO-HTMG.

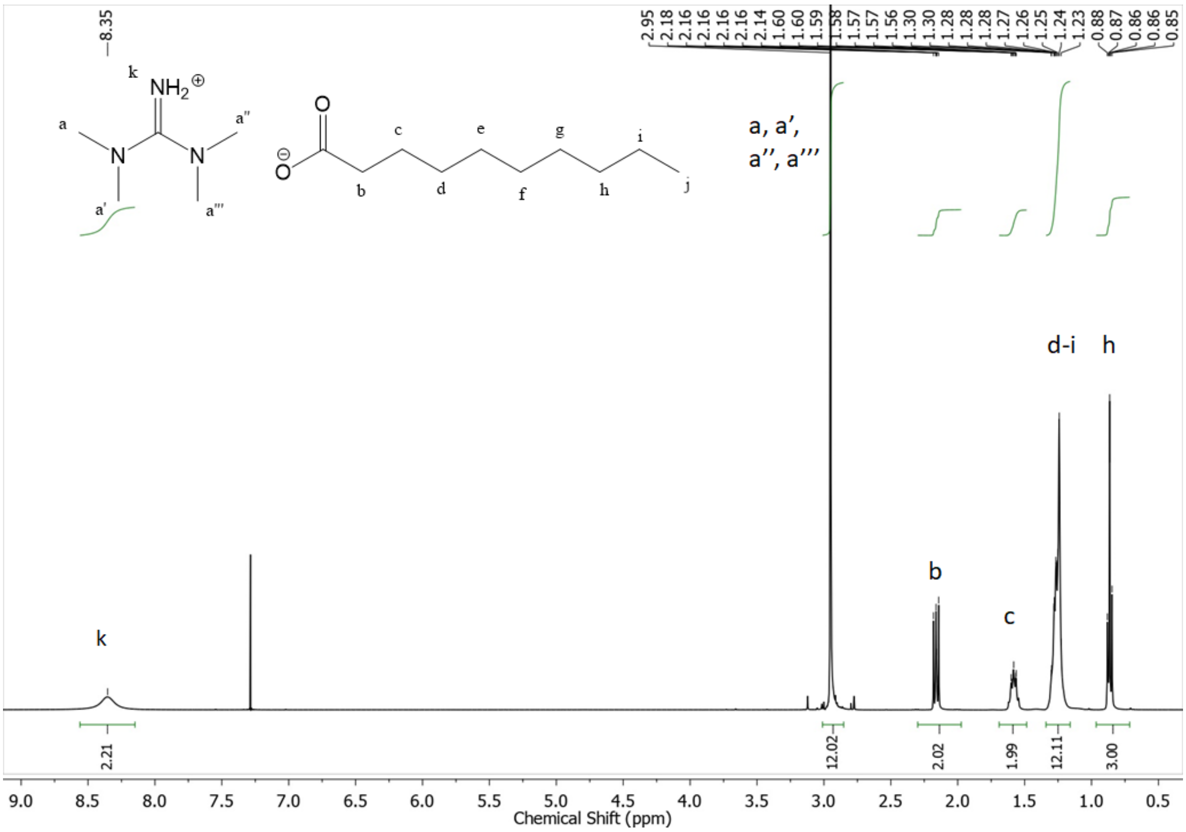

Figure S2. ^{1}H NMR of C_9CO-HTMG.

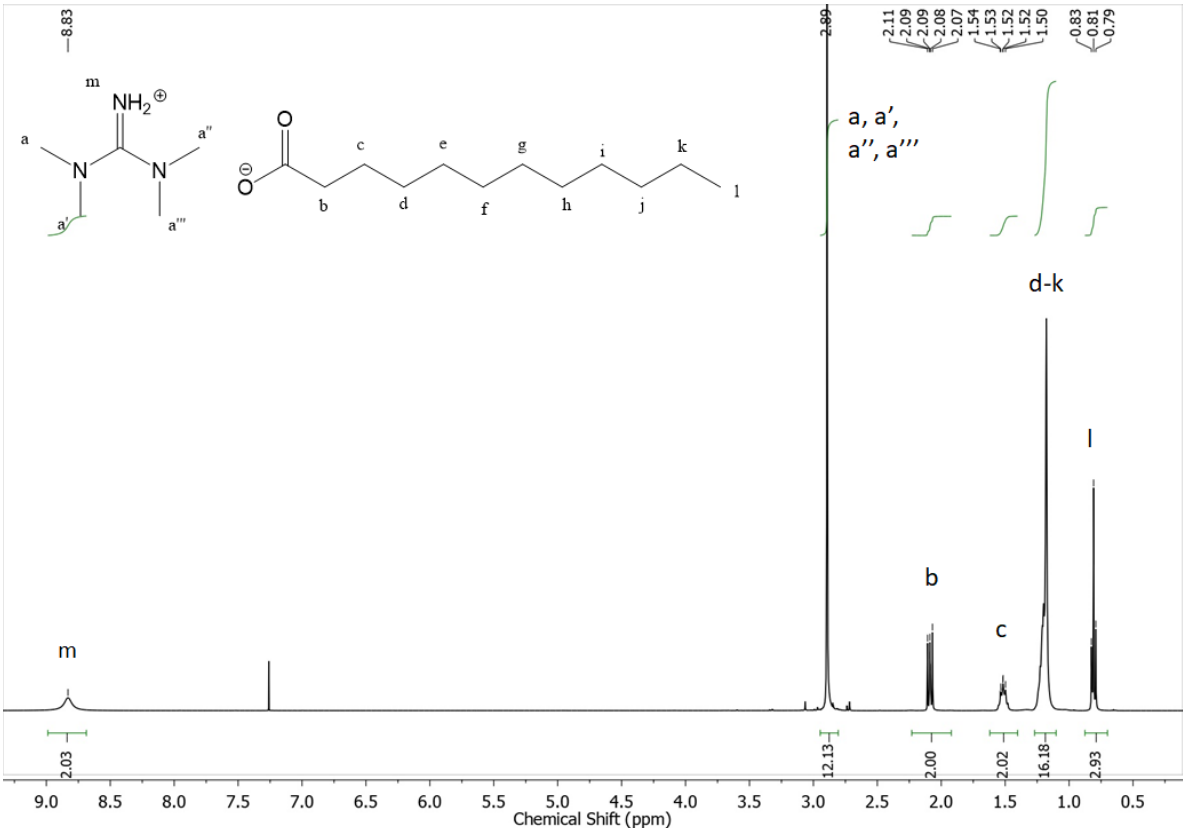

Figure S3. ^{1}H NMR of C_{11}CO-HTMG.

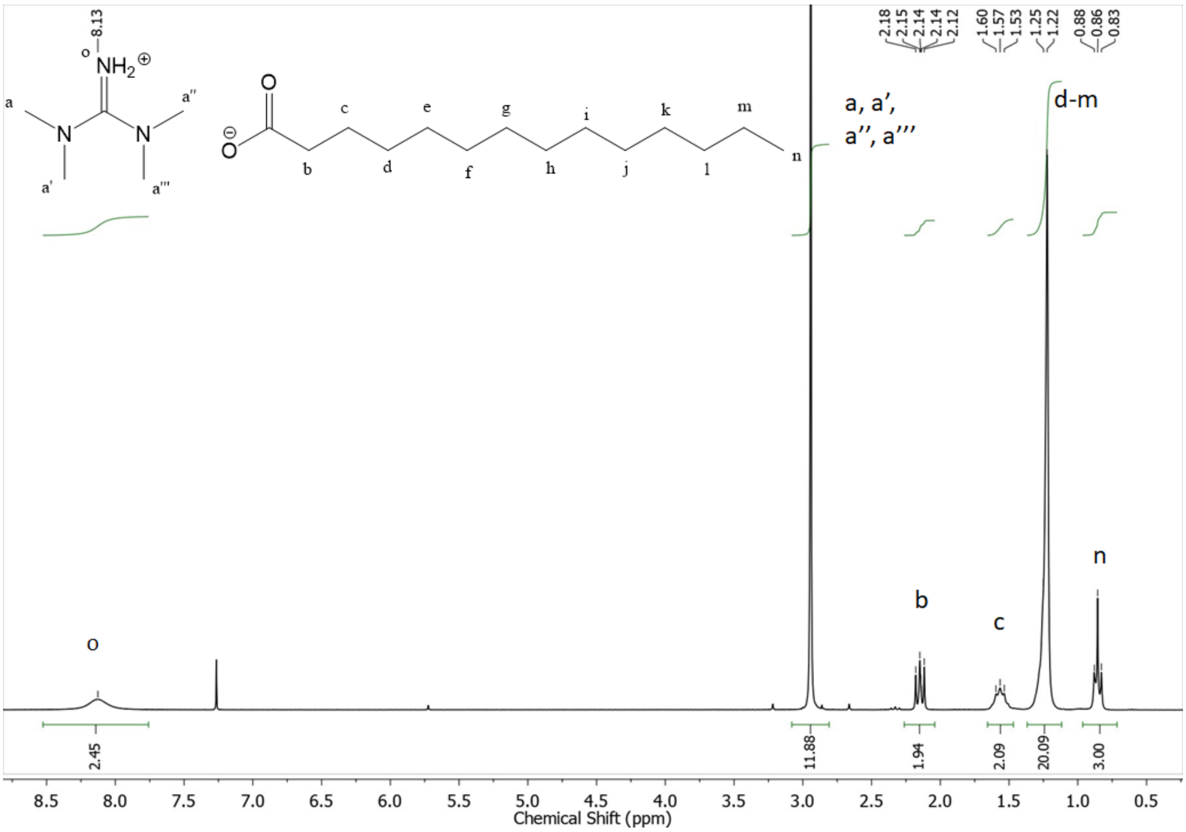

Figure S4. ^{1}H NMR of C_{13}CO-HTMG.

DSC

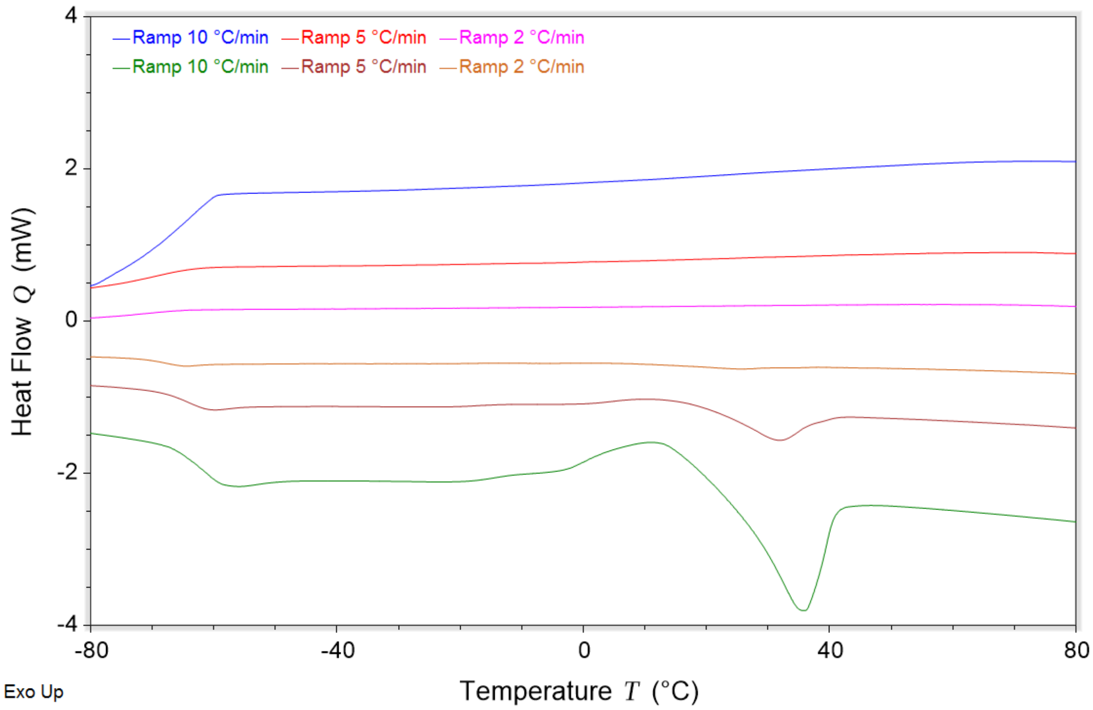

Figure S5. Differential scanning calorimetry (DSC) of compound C_7CO-HTMG at different scanning rates.

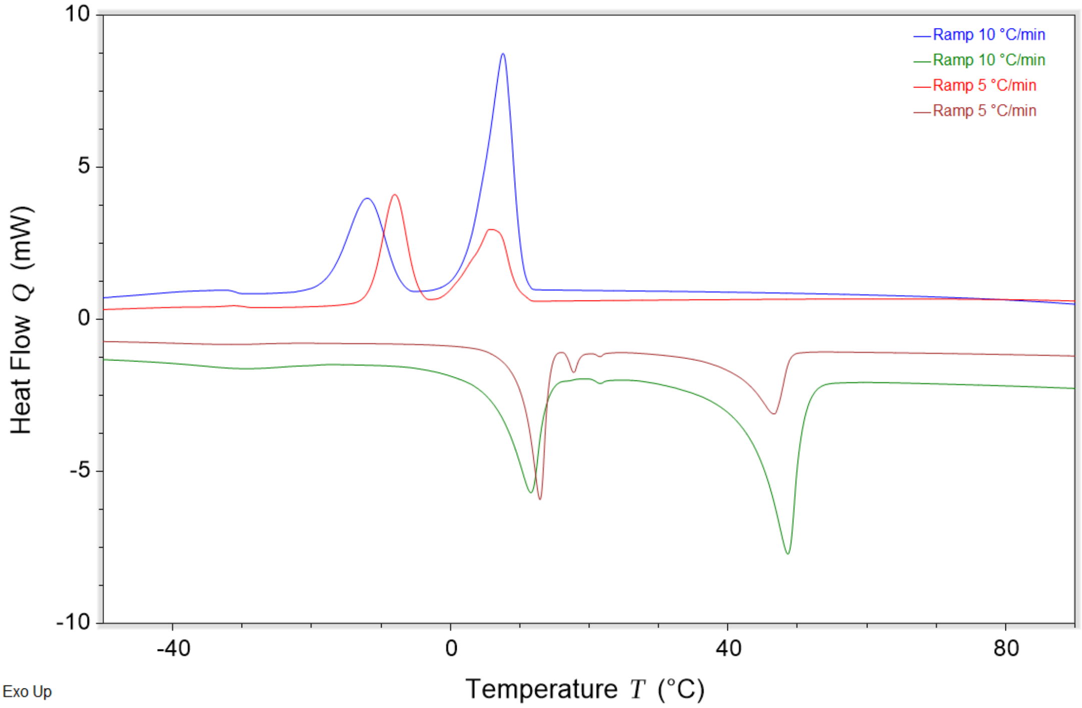

Figure S6. Differential scanning calorimetry (DSC) of compound C_9CO-HTMG at different scanning rates.

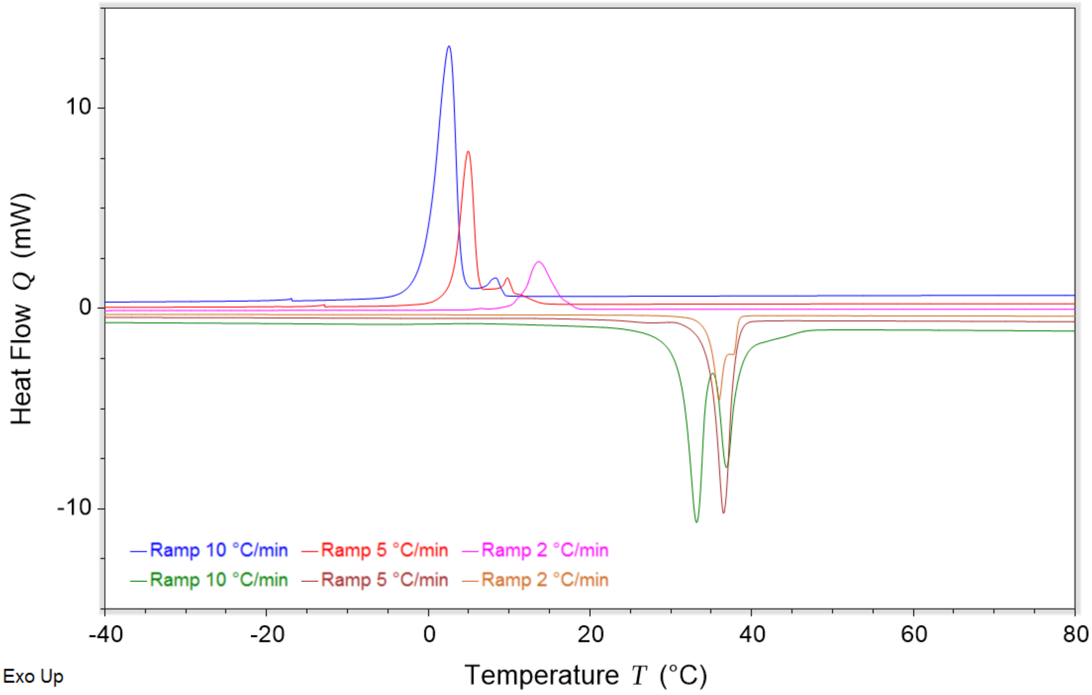

Figure S7. Differential scanning calorimetry (DSC) of compound C_{11}CO-HTMG at different scanning rates.

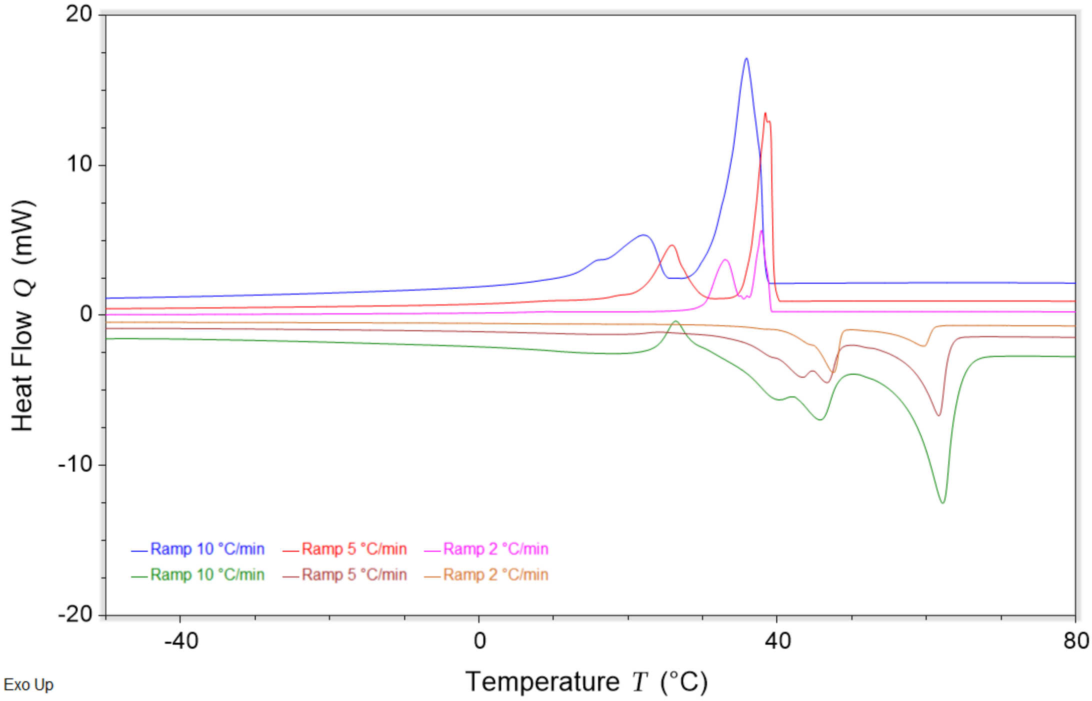

Figure S8. Differential scanning calorimetry (DSC) of compound C_{13}O-HTMG at different scanning rates.

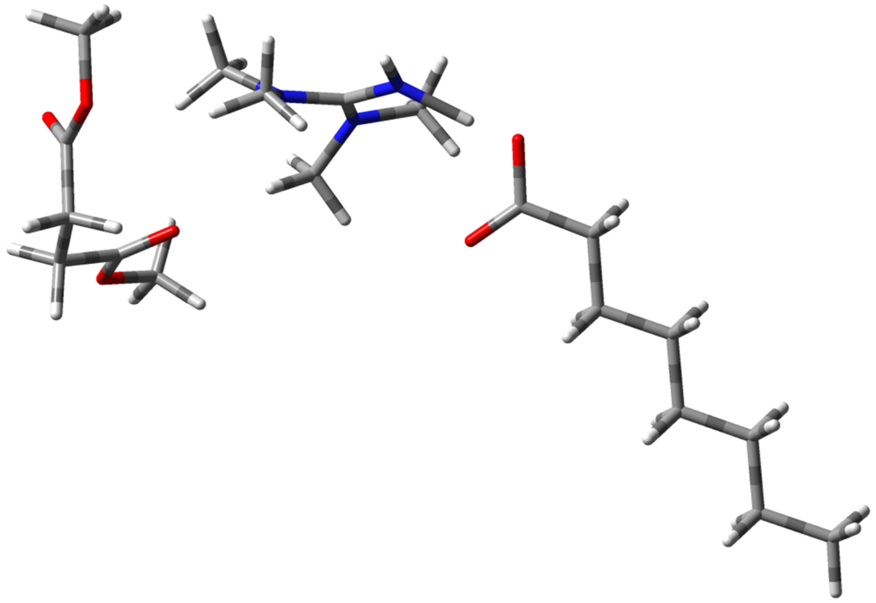

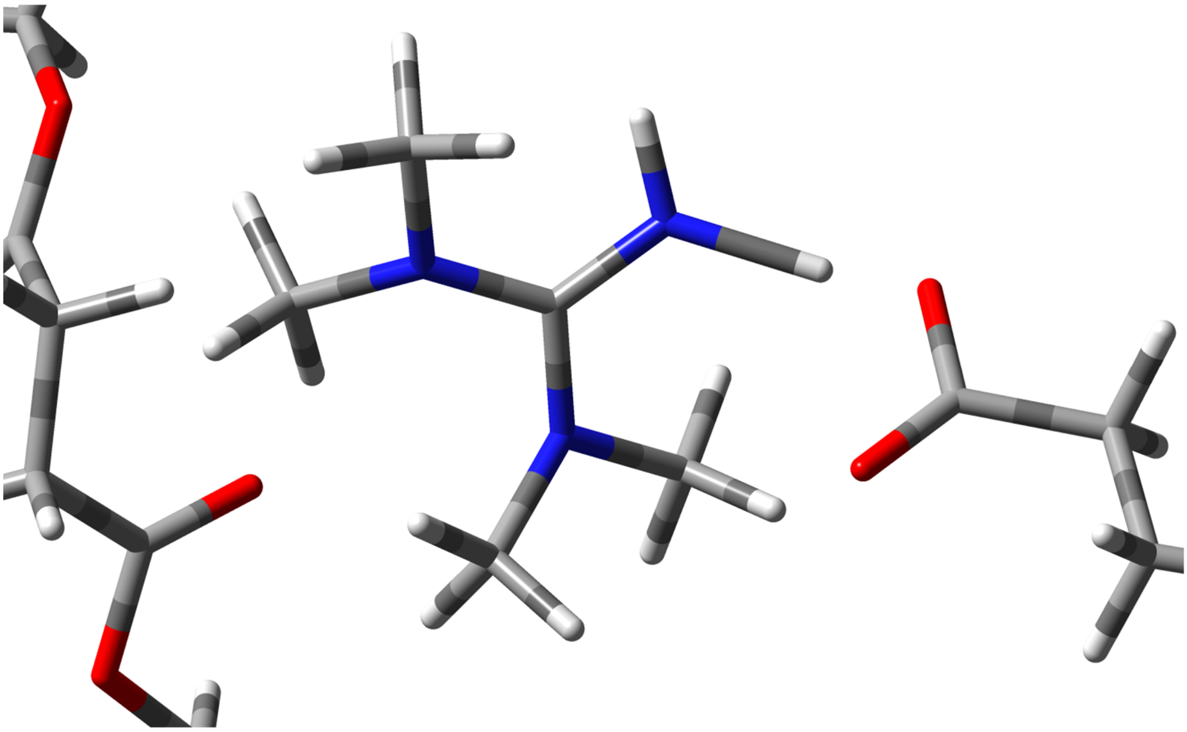

Figure S9. Top: larger picture of the 1:1:1 C_7CO-HTMG-Model of TPGS cluster. Bottom: close-up of interaction between HTMG and Model of TPGS. Comb-like hydrogen bonds of polar groups with ILs hydrogens in α position to a positively charged center are very common for ILs based on quaternary positively charged nitrogen centers.

The cluster and its components have been fully optimized and characterized as local minima. The b3lyp/6-311++g(2d,2p) level of theory has been used. Water solvent effect has been included using IEFPCM continuum model. Calculations have been performed using the Gaussian 16 Package [S1] on an Opensuse Linux Cluster based on dual Intel(R) Xeon(R) Gold 5218 CPU with SSD and 128Gb RAM. The geometry of the cluster is reported below in .xyz format.

```
67

      6          -2.794006   -1.560893    0.184860
      8          -2.176747   -0.509806    0.091644
      8          -2.222925   -2.751882    0.180435
      6          -4.302593   -1.638434    0.308115
      6          -5.010313   -0.288223    0.308318
      1          -4.526927   -2.195841    1.221265
      1          -4.662206   -2.265397   -0.512054
      6          -6.529567   -0.421478    0.423671
      1          -4.631827    0.318880    1.133580
      1          -4.757828    0.251933   -0.606712
      6          -7.254142    0.925386    0.421506
      1          -6.904068   -1.034026   -0.402754
      1          -6.778108   -0.963774    1.341586
      1          -6.879499    1.537619    1.248096
      1          -7.004691    1.467156   -0.496490
      6          -8.774066    0.799680    0.535561
      6          -9.500133    2.146336    0.531954
      1          -9.024236    0.258570    1.454001
      1          -9.149109    0.186645   -0.290552
      6         -11.019091    2.008002    0.646273
      1          -9.125126    2.758183    1.357551
      1          -9.249828    2.686223   -0.385910
```

1	-11.511935	2.981174	0.641274
1	-11.422030	1.425597	-0.184895
1	-11.296514	1.498206	1.571221
6	1.164553	-1.989255	-0.620434
7	0.777266	-1.468777	-1.828587
7	0.358782	-2.781938	0.037109
7	2.407496	-1.617192	-0.170753
6	1.051738	-0.076308	-2.176954
1	1.482376	-0.009190	-3.177502
1	0.119912	0.495645	-2.165762
1	1.736065	0.372466	-1.466392
6	-0.354379	-2.050926	-2.535022
1	-1.302399	-1.618047	-2.207595
1	-0.232641	-1.856599	-3.600549
1	-0.384385	-3.123788	-2.372277
6	3.552896	-1.504074	-1.071083
1	4.252090	-2.323838	-0.882370
1	3.231869	-1.567200	-2.104591
1	4.067957	-0.557895	-0.913481
6	2.759516	-1.812841	1.226147
1	3.130683	-2.824230	1.426218
1	3.544182	-1.106506	1.488639
1	1.900855	-1.617386	1.863893
1	-1.171399	-2.700085	0.090809
1	0.824878	-3.268999	0.791215
6	5.965211	0.642514	1.237322
6	5.365137	1.898191	1.824047
6	5.114540	2.969699	0.774425
6	4.048206	2.564197	-0.215222
1	4.447294	1.630824	2.344679
1	4.776624	3.889884	1.254444
1	6.058896	2.266893	2.581236
1	6.024022	3.214308	0.229735
8	5.950331	-0.364014	2.123709
8	4.075437	3.332149	-1.312590
6	6.553784	-1.605519	1.693726
1	7.610791	-1.455737	1.486614
1	6.419385	-2.293566	2.520634
1	6.056226	-1.978242	0.802383
6	3.074910	3.043891	-2.316113
1	2.077608	3.140013	-1.893439
1	3.231004	3.776622	-3.099748
1	3.212929	2.036618	-2.701047
8	6.416697	0.540805	0.118181
8	3.248594	1.670569	-0.043370

[S1] Gaussian 16, Revision C.01, M. J. Frisch, G. W. Trucks, H. B. Schlegel, G. E. Scuseria, M. A. Robb, J. R. Cheeseman, G. Scalmani, V. Barone, G. A. Petersson, H. Nakatsuji, X. Li, M. Caricato, A. V. Marenich, J. Bloino, B. G. Janesko, R. Gomperts, B. Mennucci, H. P. Hratchian, J. V. Ortiz, A. F. Izmaylov, J. L. Sonnenberg, D. Williams-Young, F. Ding, F. Lipparini, F. Egidi, J. Goings, B. Peng, A. Petrone, T. Henderson, D. Ranasinghe, V. G. Zakrzewski, J. Gao, N. Rega, G. Zheng, W. Liang, M. Hada, M. Ehara, K. Toyota, R. Fukuda, J. Hasegawa, M. Ishida, T. Nakajima, Y. Honda, O. Kitao, H. Nakai, T. Vreven, K. Throssell, J. A. Montgomery, Jr., J. E. Peralta, F. Ogliaro, M. J. Bearpark, J. J. Heyd, E. N. Brothers, K. N. Kudin, V. N. Staroverov, T. A. Keith, R. Kobayashi, J. Normand, K. Raghavachari, A. P. Rendell, J. C. Burant, S. S. Iyengar, J. Tomasi, M. Cossi, J. M. Millam, M. Klene, C. Adamo, R. Cammi, J. W. Ochterski, R. L. Martin, K. Morokuma, O. Farkas, J. B. Foresman, and D. J. Fox, Gaussian, Inc., Wallingford CT, 2016.

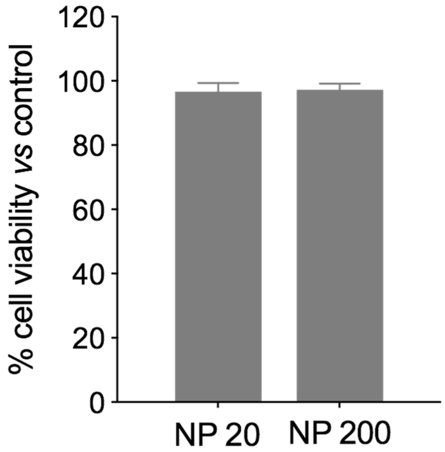

Figure S10. Cell viability of 501Mel cells treated with C_7/C_{13}CO-HTMG-TPGS /Nano. 501Mel cells (4 × 10^3 cells) were seeded in 96 well plates and, after overnight attachment, treated with C_7/C_{13}CO-HTMG-TPGS/Nano in concentrations corresponding to those loaded with IMQ (NP 20 and NP 200 correspond to C_7/C_{13}CO-HTMG-TPGS /Nano concentrations loaded with IMQ 20 and 200 ng/mL, respectively). After further 24 h, cell viability was determined by MTS assay. Data are presented as means ± SD of three independent experiments.
